# Ethnic Differences in Pregnancy Complications Among Women with Gestational Diabetes

**DOI:** 10.3390/medsci14040482

**Published:** 2026-08-14

**Authors:** Tiziana Filardi, Paola Galoppi, Stefania Gorini, Ilenia Mappa, Giuseppe Rizzo, Susanna Morano

**Affiliations:** 1 Department for the Promotion of Human Sciences and Quality of Life, San Raffaele Roma Open University, Via di Val Cannuta 247, 00166 Rome, Italy; stefania.gorini@uniroma5.it; 2 The Department of Maternal and Child Health and Urological Sciences, Sapienza University, Viale Regina Elena 324, 00161 Rome, Italy; paola.galoppi@uniroma1.it (P.G.); ilenia.mappa@uniroma1.it (I.M.); giuseppe.rizzo@uniroma1.it (G.R.); 3 Laboratory of Cardiovascular Endocrinology, IRCCS San Raffaele, Via di Val Cannuta 247, 00166 Rome, Italy; 4 Department of Experimental Medicine, Sapienza University, Viale Regina Elena 324, 00161 Rome, Italy; susanna.morano@uniroma1.it

**Keywords:** gestational diabetes, GDM, ethnicity, ethnic differences, racial differences, pregnancy outcomes, perinatal outcomes, delivery, GDM complications, GDM outcomes

## Abstract

**Background/Objectives**: Gestational diabetes mellitus (GDM) is a metabolic disorder associated with an amplified risk of unfavorable pregnancy outcomes. This study aimed to compare clinical characteristics and pregnancy outcomes among GDM patients belonging to different ethnic groups. **Patients and Methods**: This retrospective analysis included *n* = 466 women with GDM (*n* = 167 Asian; *n* = 299 European). The impact of ethnicity on pregnancy outcomes was evaluated using multivariable logistic regression models, adjusting for confounding factors. **Results**: Asians were younger than Europeans (*p* < 0.001) and more frequently multiparous (*p* = 0.003). Fasting glucose at OGTT was higher in Asians, as well as HbA1c values at the end of pregnancy (*p* = 0.010 and *p* = 0.001, respectively). Asian ethnicity was associated with an increased likelihood of insulin therapy (OR = 3.36 [1.72–6.58] 95% CI, *p* < 0.001), in the adjusted model. The gestational week of delivery was lower in Asians compared to Europeans (*p* = 0.007). The Asian group had a higher occurrence of cesarean section (*p* = 0.048) and emergency cesarean section (*p* = 0.006) compared to the European group. Delivery complications were more frequent in the Asian cohort, adjusting for confounders (OR = 2.19 [1.11–4.32] 95% CI, *p* = 0.023). Newborns of the Asian group were more frequently small for gestational age (SGA) in adjusted models (OR = 2.36 [1.08–5.15] 95% CI, *p* = 0.031). **Conclusions**: Worse GDM outcomes were observed in the Asian cohort compared to the European cohort. Identifying risk factors for GDM-related complications is crucial for developing tailored interventions and reducing the overall disease burden.

## 1. Introduction

Gestational diabetes mellitus (GDM) is a widespread metabolic disorder associated with an increased risk of unfavorable obstetric outcomes [1,2,3]. GDM adversely affects the long-term cardiometabolic profile of both the mother and the offspring, enhancing the risk for future type 2 diabetes (T2D), metabolic syndrome and cardiovascular events [4,5,6,7,8,9]. Specific ethnic groups, including the South and the East Asians, the Hispanics, the Africans, the Pacific Islanders, the Indigenous Australians and the Native Americans have higher risk of GDM compared to the Caucasians [10,11], likely owing to genetic, lifestyle and socio-cultural factors [12,13]. In the last decades, the intensification of the migratory flows from high-risk geographic areas to low-risk countries has stimulated a growing body of research aimed at exploring ethnic disparities in GDM [14]. A variety of features of GDM have displayed profound ethnic differences. Asides from prevalence, clinical characteristics, patterns of hyperglycemia at diagnosis, as well as treatment adherence were reported to significantly vary between ethnic groups [13]. Regarding maternal and perinatal outcomes, current research has yielded quite inconsistent results. Several studies have highlighted relevant racial disparities in perinatal outcomes of GDM, with higher rates of some adverse events in non-Caucasian ethnicities, such as preterm birth, caesarean delivery, large for gestational age (LGA), macrosomia and hypoglycemia [3,15,16,17,18,19,20]. Conversely, other authors have not confirmed significant effects of ethnicity on pregnancy outcomes, postulating the ‘healthy migrant paradox’, indirect evidence of a more intensive care given to individuals with social disadvantage, such as migrants belonging to high-risk ethnicities, in some host countries [21,22]. The identification of key factors associated with maternal and neonatal complications is fundamental to tailor interventions aimed at containing the burden of GDM in modern multicultural countries.

We aimed to investigate the disparities in obstetric and neonatal outcomes between European and Asian women with GDM in a single Italian Diabetes Centre and to identify any factors associated with pregnancy complications of GDM.

## 2. Patients and Methods

This is a retrospective cohort study, which involved 466 women with singleton pregnancy and diagnosis of GDM. Participants were recruited between January 2016 and June 2024 at the outpatient clinic of Endocrinology and Diabetology, Department of Experimental Medicine, Policlinico Umberto I, “Sapienza” University of Rome. Diagnosis of GDM was defined according to current Italian guidelines [23,24,25]. The study was conducted in accordance with the Declaration of Helsinki, and the protocol was approved by the Hospital Ethics Committee of Policlinico Umberto I “Sapienza” University of Rome (project number 3830, protocol code 2627/15, date of approval 22 October 2015). All the enrolled subjects provided written informed consent.

Women with the following characteristics were excluded: age < 18 years, twin pregnancy, pre-gestational diabetes, relevant maternal conditions affecting pregnancy outcomes (alcohol abuse, psychiatric diseases, serious infectious, renal, liver and heart maternal diseases).

Demographic data (age and ethnicity), family history (diabetes, dyslipidemia, hypertension, and cardiovascular disease, malformations), health behavior information (smoking, alcohol consumption), reported weight and body mass index (BMI) before pregnancy, obstetric history (gestational age, estimated delivery date, previous pregnancies and/or miscarriages, previous GDM), personal medical history and pharmacological therapy information were collected. Maternal ethnicity was inferred using the patient’s country of birth as extracted from medical records at first visit. At each pregnancy visit after GDM diagnosis, anthropometric, laboratory and fetal ultrasound parameters, including bi-parietal diameter (BPD), head circumference (HC), abdominal circumference (AC), femur length (FL), humerus length (HL), estimated fetal weight (EFW) and fetal weight percentile were collected. During the first visit for GDM, all patients were provided with a standardized nutritional plan based on Mediterranean diet.

### 2.1. Outcome Measures

Delivery data and adverse pregnancy outcomes were analyzed. Specifically, a composite of delivery complications, including preterm birth (<37 weeks), operative vaginal delivery, emergency cesarean section was evaluated.

Fetal and neonatal outcomes were: preterm birth; large for gestational age infants (LGA, birth weight > 90th percentile); macrosomia (birth weight > 4000 g); small for gestational age infants (SGA, birth weight ≤ 10th percentile); low birth weight (weight < 2500 g). For SGA and LGA infant definitions the INeS charts were used [26,27]. Percentiles from INeS charts are already adjusted for gestational week at delivery and for fetal sex. A composite of adverse perinatal outcomes, consisting of at least one of the following perinatal complications: neonatal death, admission to neonatal intensive care unit, abnormal Apgar score (<7 at 1 min or 5 min), respiratory complications (oxygen therapy, assisted ventilation, intubation, respiratory acidosis, respiratory distress), phototherapy for neonatal jaundice, hypoglycemia, and hypocalcemia, was also analyzed. Outcomes of interest were selected due to their consistent association with gestational diabetes and were grouped in line with previously published research [3].

### 2.2. Statistical Analysis

Continuous variables are reported as mean ± standard deviation (SD), whereas categorical variables are shown as percentages or frequencies. Normality of continuous variables was verified using the Kolmogorov-Smirnov test. Inter-group comparisons for normally distributed continuous data were performed via the independent-samples *t*-test; otherwise, the Mann-Whitney U test was employed. For continuous variable, differences between group are presented as Estimated Mean Differences with 95% confidence intervals (95% CI). Proportions for categorical data were compared using the Chi-square test or Fisher’s exact test, as appropriate, and are reported as percentages, with effect sizes expressed as Odds Ratios (OR) with corresponding 95% CI. To evaluate differences in continuous outcomes between the two cohorts while adjusting for covariates, linear models were applied. Furthermore, multivariable logistic regression models were built to investigate the association between the primary variables of interest, controlling for potential confounders. The multivariable models were restricted to the main primary covariates known to be associated with the outcome of interest, according to current evidence from literature. Covariates not showing significant association in the univariable analysis were excluded from the multivariable model. Specifically, the multivariable model for insulin therapy included the following covariates: age, ethnicity, pre-pregnancy BMI, parity, history of GDM, weight gain, fasting plasma glucose at oral glucose tolerance test, and pregnancy week at diagnosis of GDM. The logistic model for delivery complication was implemented with age, ethnicity, parity, pre-pregnancy BMI, hypertension, weight gain, pregnancy week at diagnosis of GDM. The multivariable model for SGA included age, ethnicity, parity and gestational hypertension. The study sample fulfilled the recommended Events-Per-Variable (EPV) criterion across all performed analyses. To avoid overfitting and ensure adequate statistical power, the number of covariates included in each multivariable model was strictly constrained based on the frequency of the least frequent outcome (maintaining an EPV ratio equal or higher than 10–15). Missing data were addressed using a complete-case analysis approach, in which observations with missing values for any of the variables included in the multivariable models were excluded from the final analysis. Given that the primary statistical hypotheses were planned a priori, formal adjustments for multiple testing were not applied. Instead, to avoid sole reliance on binary *p*-value thresholds, statistical evaluation focused on the magnitude and precision of observed effects. Accordingly, standardized effect sizes along with their corresponding 95% confidence intervals (95% CI) are reported for all primary comparisons.

Statistical significance was defined as a *p*-value < 0.05. All analyses were conducted using IBM SPSS Statistics software, version 23.0 (IBM Corp., Armonk, NY, USA).

## 3. Results

A total of 466 eligible women with positive diagnostic test for GDM were involved in the analysis. Of these, 167 were Asian women and 299 were European women. The Asian group included 117 women from South Asia and 50 women from East Asia. All the Asian women included in this study were migrants.

### 3.1. Risk Factors and Clinical Characteristics

GDM risk factors across study groups according to geographic origin are presented in Table 1. The South and East Asians were significantly younger than the Europeans (*p* < 0.001). Gestational week at first visit and pre-pregnancy BMI were not different among groups (Table 1). The prevalence of first-degree family history of T2D was higher in the South Asian group compared to the East Asian group (*p* = 0.017), as well as compared to the European group (*p* = 0.002). Women from East and South-East Asia were more frequently multiparous (Table 1).

Baseline characteristics and laboratory parameters of the overall Asian group and the European group are shown in Table 2. Asian women were significantly younger than European women (31.9 ± 5.3 vs. 35.8 ± 5.1 years, *p* < 0.001). Systolic blood pressure was lower in the Asian group than in the European group, although this finding was not confirmed after adjusting for age (109.4 ± 1.3 vs. 112.4 ± 0.9 mmHg, *p* = 0.057).

Multiparity was more frequent among Asian women (76.4% vs. 62.5%, *p* = 0.003). Fasting plasma glucose at OGTT was significantly higher in Asians than in Europeans (92.3 ± 11.9 vs. 88.7 ± 12.1 mg/dL, *p* = 0.010). Asians had higher HbA1c values at the end of pregnancy (5.5 ± 0.4 vs. 5.3 ± 0.5%, *p* = 0.001) and were more likely to start insulin therapy compared to European women (53.7% vs. 35.1%, *p* < 0.001).

In the multivariable logistic regression model, Asian ethnicity (OR = 3.36 [1.72–6.58] 95% CI, *p* < 0.001), pre-gestational BMI (OR = 1.06 [1.01–1.11] 95% CI, *p* = 0.030), fasting plasma glucose at OGTT (1.05 [1.02–1.08] 95% CI, *p* = 0.010) and history of previous GDM (OR = 3.30 [1.26–8.60] 95% CI, *p* = 0.015) were found to be independently associated with insulin therapy during pregnancy (Table 3). Asian ethnicity was therefore associated with a three-fold increase in the likelihood of insulin requirement, independent of age, parity, history of previous GDM, pre-pregnancy BMI, weight gain, fasting glucose at OGTT and gestational week at GDM diagnosis.

Fetal ultrasound parameters in the third trimester of gestation were not different between the two groups.

### 3.2. Pregnancy and Perinatal Outcomes

Delivery data are presented in Table 4. The gestational week of delivery was lower in Asian women compared to European women (37.7 ± 2.29 vs. 38.4 ± 1.95 weeks, *p* = 0.007). Asian group had a higher rate of cesarean section (74.3% vs. 63.0%, *p* = 0.048) and emergency cesarean section (30.1% vs. 16.4%, *p* = 0.006) compared to the European group.

The prevalence of the composite of delivery complications (preterm birth, assisted vaginal delivery, emergency cesarean section) was higher in Asians (44.6% vs. 27.3%, *p* = 0.003). In the multivariable logistic regression model, Asian ethnicity was associated with increased likelihood of delivery complications, regardless of age, pre-pregnancy BMI, parity, weight gain, gestational week at GDM diagnosis, and presence of gestational arterial hypertension (OR = 2.19 [1.11–4.32] 95% CI, *p* = 0.023) (Table 5).

A higher prevalence of low birth weight (weight < 2500 g) (20.2% vs. 11.5%, *p* = 0.045) and SGA (17.6% vs. 8.9%, *p* = 0.030) was observed in infants born to Asian mothers, compared to European mothers (Table 4). In the multivariable logistic regression analysis, Asian ethnicity was significantly associated with an increased likelihood of SGA infants, adjusting for confounding factors, such as age, parity, gestational arterial hypertension (OR = 2.36 [1.08–5.15] 95% CI, *p* = 0.031) (Table 6). The prevalence of macrosomia and LGA was comparable between the two groups (Asians 2.9% vs. Europeans 4.9%, *p* = 0.417 and Asians 6.9% vs. Europeans 10.6%, *p* = 0.303, respectively) (Table 4). The composite adverse perinatal outcome was not significantly different between the two groups (Asians 22.9% vs. Europeans 18.3%, *p* = 0.361) (Table 4). Data on perinatal complications refer to 21 newborns of Asian mothers and 43 newborns of European mothers.

## 4. Discussion

In this retrospective study, Asian and European women with GDM were compared for both clinical features and pregnancy-related outcomes, in a single Italian center. The two groups had some relevant differences in risk factors and baseline characteristics. Particularly, women from Asia were younger than European women, and had a lower rate of nulliparity. These findings confirmed previous observations suggesting distinctive socio-cultural trends in gestation among different ethnicities [16,21,22,28]. Previous miscarriages, prior GDM and macrosomia were comparable between the two cohorts. Differently from other reports, pre-pregnancy BMI did not differ between European and Asian patients with GDM [15,16,17,18,20]. In line with our findings, Caputo et al. reported similar pre-gestational BMI in both the Italian and the Asian population with GDM [21]. There is consistent evidence that the relative contribution of BMI as a risk factor for GDM is largely modulated by ethnicity [29,30]. Nevertheless, migration trends to regions with high obesity rates may drive lifestyle modifications that increase the prevalence of overweight and obesity in migrant populations, likely narrowing the disparities [31,32,33].

Asian women had higher values of fasting plasma glucose at OGTT than European women. Prior research has reported ethnic differences in glycemic profiles during OGTT, although the results are conflicting. Overall, both fasting and post-load glucose at OGTT were higher in non-Caucasian women compared with Caucasian women in most studies [16,34,35,36]. Of note, higher fasting glucose at OGTT have been consistently linked to poorer pregnancy outcomes in GDM [21,37,38,39]. In contrast, Read et al. observed lower fasting glucose values at OGTT in Asian women compared with Caucasian women [16]. In a prior study, in which we compared pregnancy outcomes in high risk and low risk ethnicities, fasting and post-load glucose values did not differ between the study groups [28]. Nevertheless, it should be highlighted that the observed results applied to different populations, which do not entirely match with the population involved in the present study.

In this study, third trimester HbA1c had higher levels in Asian women than in European women. Although the role of HbA1c as a reliable predictor of unfavorable outcomes in GDM-complicated pregnancies is not yet fully established, emerging evidence suggested a possible link between specific HbA1c thresholds across different stages of pregnancy and gestational complications [40,41,42]. Additional research is required to fully elucidate whether HbA1c can predict both GDM and its associated adverse outcomes.

In this study, Asian ethnicity was associated with an increased probability of insulin use, independently of pre-gestational BMI and history of previous GDM, confirming previous findings [21,22,43,44,45]. Whether the increased insulin requirement is attributable to lifestyle factors or distinct underlying mechanisms in GDM pathophysiology remains to be fully elucidated. The amount and the quality of carbohydrate consumption differ substantially across diverse cultural groups [34]. Although all the patients had been provided with a standardized nutritional plan, long-term adherence to dietary recommendations and potential shifts from native eating habits were not evaluated. It is worth noting that insulin initiation may be also influenced by physician practice patterns, language barriers, and healthcare access. In addition, maternal education, household income, physical activity, and timing of prenatal care initiation may also substantially contribute to the observed ethnic differences in this outcome. Of note, language barriers and low health literacy might consistently limit the understanding of prescriptions, further affecting patient compliance [46]. Rather than applying a rigid dietary approach, nutritional advise should incorporate culturally sensitive plans that accommodate non-native eating habits while encouraging Mediterranean dietary principles. To address the complex needs of migrant populations facing language and cultural barriers, healthcare systems might implement dedicated multidisciplinary care pathways supported by specialized professionals.

Asian women delivered at a significantly earlier gestational age than European women. Furthermore, the Asian cohort showed substantially higher rates of cesarean sections compared to their European counterparts. We had previously reported that women with GDM belonging to high-risk ethnicities, including Asian ethnicity, had lower gestational age at delivery than low-risk women [28]. Other authors reported a higher risk of preterm birth and cesarean delivery among migrant women in comparison to Italian mothers [18,22]. Accordingly, a significantly elevated prevalence of composite delivery complications was observed in the Asian cohort independently of confounders. It may be speculated that this occurrence might be linked to the adoption of a more aggressive clinical approach when facing significant risk factors, such as poor glycemic control, or even socioeconomic disadvantages, to minimize adverse outcomes. However, no data regarding clinician decision-making, induction protocols, or institutional management pathways were available to support this hypothesis. Of note, a higher rate of emergency caesarean section was observed in Asian mothers. According to findings by Von Katterfeld et al., women born abroad, comprising a large proportion of Asian individuals, had higher rate of emergency cesarean sections than their Australian-born counterparts, independently of GDM diagnosis [19]. Similarly to the present study, this trend did not aligned with a higher prevalence of typical predisposing factors for emergency cesarean sections, including pre-eclampsia or LGA infants. Unmeasured confounding variables might account for the increased risk for emergency Caesarean sections in this population.

Asian newborns were more frequently SGA compared to European newborns, even after controlling for maternal confounders such as age, parity, and gestational hypertension. Conversely, the two cohorts displayed comparable rates of fetal macrosomia and LGA infants. In a recent study, Asian women with GDM showed an increased risk for SGA and a lower risk for LGA compared with White individuals [17]. The same authors had previously demonstrated a more frequent occurrence of SGA infants in Asian women than in White women [18]. In the present study, an increased incidence of relevant risk factors for SGA, such as hypertension and preeclampsia was not observed [47,48].

Regarding perinatal complications, no significant difference was observed in the composite adverse perinatal outcome between the cohorts, likely owing to the limited data on newborns.

This study has several limitations. Firstly, its retrospective design precludes establishing causal relationships. Inferring ethnicity from patients’ country of birth may not directly reflect true ethnic backgrounds, introducing potential misclassification bias—particularly among second-generation immigrants or individuals originating from ethnically heterogeneous countries. Another major limitation lies in the heterogeneity of the Asian cohort, which encompasses women from various regions of Asia. These populations may differ considerably in genetic background, body composition, dietary habits, socioeconomic characteristics, migration history, and risk factors for GDM. However, the small sample size of the Asian cohort provided insufficient statistical power to perform subgroup analyses for pregnancy outcomes. The primary objective of this study was to compare clinical characteristics, as well as pregnancy and perinatal outcomes, between two broader cohorts defined by distinct ethnicity-related GDM risk levels. The subgroups were therefore combined into a single Asian cohort for the primary multivariable analyses to ensure robust estimate stability. In addition, the limited data on newborns precludes drawing firm conclusions regarding neonatal complications. Of note, as formal corrections for multiple comparisons were not performed, unadjusted *p*-values carry a potential risk of inflated Type I error. Future studies including independent subgroups are mandatory to confirm these findings. The multivariable models were restricted to the main primary covariates known to be associated with the outcome of interests, likely missing some relevant additional confounders. A further limitation lies in the reliance on composite outcomes, which may mask differences in the weight and clinical relevance of single neonatal or maternal events. Specifically, composite maternal and neonatal outcomes encompass events with distinct clinical severity and pathophysiological mechanisms, and their interpretation warrants appropriate caution. In addition, data regarding the main social determinants of health, such as income, education, environment factors were not available, as well as information about the duration of residence in Italy, language proficiency, and adherence to dietary recommendations. Given that ethnicity served as a primary variable of interest, these unmeasured variables may account for part of the observed disparities. Consequently, ethnic differences identified in this study must be interpreted with caution and treated as associations rather than direct causal relationships. Even data on previous cesarean section and self-monitoring of blood glucose were unavailable. Finally, the single-center, retrospective design of this study, conducted at a tertiary diabetes center, may have introduced referral bias. Specifically, referral patterns may vary according to ethnicity, potentially influencing the overall case mix and clinical severity. This study reflects clinical management within one Italian center operating under specific guidelines. Consequently, the external validity of the results may be limited in settings with alternative protocols for insulin therapy and obstetric management.

Despite these limitations, the strengths of this study include the large sample size, the collection of clinical data across multiple stages of pregnancy and the availability of delivery data. In addition, the present study provides interesting directions for future research. Further studies are warranted to reveal the key factors that may drive ethnic disparities in glycemic control and pregnancy outcomes.

## 5. Conclusions

In this retrospective study, the comparison between Asian and European ethnicity revealed significant differences in some pregnancy outcomes of GDM. Whether these disparities are primarily driven by genetic factors and distinct pathophysiological features of GDM, or even by socioeconomic and cultural differences among diverse ethnic groups remains to be elucidated.

## Figures and Tables

**Table 1:** GDM risk factors and differences among groups according to the area of origin.

	South Asian Group (*n* = 117)	East Asian Group (*n* = 50)	European Group (*n* = 299)	Mean Difference/Odds Ratio	95% CI	*p*-Value
Age (years)	31.3 ± 5.2	32.8 ± 5.3	35.8 ± 5.0	−4.51 −2.94 −1.57	−5.91; −3.11 −4.59; −1.29 −3.49; 0.36	<0.001 * <0.001 ^†^ 0.137 ^††^
Gestational age 1st visit (wks)	28.4 ± 5.3	26.4 ± 5.9	27.4 ± 5.5	0.96 −0.99 1.95	−0.55; 2.47 −2.79; 0.78 −0.132; 4.04	0.296 * 0.388 ^†^ 0.072 ^††^
Pre-gestational BMI (kg/m^2^)	26.5 ± 4.1	25.1 ± 6.1	26.7 ± 6.7	−0.25 −1.64 1.39	−2.43; 1.92 −4.02; 0.74 −1.49; 4.27	0.959 * 0.238 ^†^ 0.494 ^††^
T2D Family history (%)	55.8	36.1	38.1	2.07 0.92 2.24	1.29; 3.30 0.52; 1.64 1.16; 4.33	0.002 * 0.785 ^†^ 0.017 ^††^
Prior GDM (%)	17.9	14.5	10.2	1.95 1.52 1.28	1.02; 3.73 0.68; 3.39 0.53; 3.09	0.042 * 0.304 ^†^ 0.578 ^††^
Multiparity (%)	75.5	77.8	62.5	1.83 2.08 0.88	1.09; 3.11 1.10; 3.94 0.41; 1.88	0.024 * 0.025 ^†^ 0.745 ^††^

BMI: body mass index; T2D: type 2 diabetes; GDM: gestational diabetes mellitus. * *p*-value South Asian group vs. European group. ^†^ *p*-value East Asian group vs. European group. ^††^ *p*-value South Asian vs. East Asian group.

**Table 2:** Clinical and laboratory parameters of the study population.

	Asian Group (*n* = 167)	European Group (*n* = 299)	Mean Difference/ Odds Ratio	95% CI	*p*-Value
Age (years)	31.9 ± 5.3	35.8 ± 5.1	−3.89	−4.86; −2.90	<0.001
Gestational age 1st visit (wks)	27.6 ± 5.5	27.4 ± 5.5	0.16	−0.89; 1.22	0.765
Pre-gestational BMI (kg/m^2^)	25.7 ± 5.0	26.7 ± 6.7	−1.03	−2.38; 0.32	0.134
Overweight/obesity (%)	57.0	47.5	1.47	0.91; 2.36	0.112
Gestational weight gain (kg)	10.2 ± 5.3	9.8 ± 6.1	0.39	−0.92; 1.72	0.551
T2D Family history (%)	47.5	38.1	1.47	0.99; 2.17	0.052
Prior GDM (%)	16.1	10.2	1.70	0.97; 2.99	0.063
Multiparity (%)	76.4	62.5	1.94	1.26; 2.99	0.003
Prior miscarriages (%)	33.3	33.4	0.99	0.66; 1.50	0.980
SBP (mmHg)	109.4 ± 1.3	112.4 ± 0.9	−3.05	−6.19; 0.09	0.057 ^†^
DBP (mmHg)	70.3 ± 9.4	71.5 ± 9.1	−1.18	−3.34; 0.99;	0.286
FPG at OGTT (mg/dL)	92.3 ± 11.9	88.7 ± 12.1	3.65	0.89; 6.42	0.010
1-h PG at OGTT (mg/dL)	177.8 ± 30.3	176.2 ± 31.5	1.60	−5.69; 8.89	0.666
2-h PG at OGTT (mg/dL)	153.7 ± 28.5	149.9 ± 34.9	3.73	−4.01; 11.47	0.344
Third trimester FPG (mg/dL)	87.7 ± 13.1	85.3 ± 13.5	2.45	−0.43; 5.33	0.095
HbA1c at diagnosis (%)	5.4 ± 0.5	5.5 ± 1.9	−0.11	−0.54; 0.32	0.610
Third trimester HbA1c (%)	5.5 ± 0.4	5.3 ± 0.5	0.20	0.09; 0.32	0.001
First trimester FPG (mg/dL)	96.6 ± 14.2	93.2 ± 15.9	3.46	−4.94; 11.86	0.412
Insulin therapy (%)	53.7	35.1	2.14	1.45; 3.15	<0.001
Hypertension/Preeclampsia (%)	7.3	11.4	0.61	0.31; 1.21	0.157

CI: confidence interval; wks: weeks; BMI: body mass index; T2D: type 2 diabetes; GDM: gestational diabetes mellitus; SBP: systolic blood pressure; DBP: diastolic blood pressure; FPG: fasting plasma glucose; OGTT: oral glucose tolerance test; PG: plasma glucose; HbA1c: glycated hemoglobin. ^†^ Adjusted for age.

**Table 3:** Insulin therapy: multivariable regression analysis.

Variable	OR	95% CI	*p*-Value
Age	1.05	0.98–1.11	0.134
Multiparity	0.71	0.36–1.40	0.321
Asian ethnicity	3.36	1.72–6.58	<0.001
Pre-pregnancy BMI	1.06	1.01–1.11	0.030
Prior GDM	3.30	1.26–8.60	0.015
Weight gain	0.99	0.94–1.05	0.838
FPG at OGTT	1.05	1.02–1.08	0.010
Week at GDM diagnosis	0.95	0.89–1.00	0.069

OR: Odds ratio; CI: confidence interval; BMI: body mass index; GDM: gestational diabetes mellitus; FPG: fasting plasma glucose; OGTT: oral glucose tolerance test.

**Table 4:** Delivery and perinatal outcomes.

	Asian Group (*n* = 167)	European Group (*n* = 299)	Mean Difference/ Odds Ratio	95% CI	*p*-Value
Gestational age at birth (wks)	37.7 ± 2.29	38.4 ± 1.95	−0.69	−1.19; −0.19	0.007
Preterm birth (%)	19.2	11.4	1.85	0.94; 3.60	0.068
Cesarean section (%)	74.3	63.0	1.70	1.0; 2.88	0.048
Emergency cesarean section (%)	30.1	16.4	2.19	1.24; 3.88	0.006
Operative vaginal delivery (%)	1.0	1.6	0.60	0.06; 5.86	0.650
Delivery complications ^†^ (%)	44.6	27.3	2.14	1.28; 3.60	0.003
Apgar 1 min or 5 min < 7 (%)	11.5	4.8	2.61	0.93; 7.30	0.060
Umbilicar artery pH	7.28 ± 0.08	7.28 ± 0.12	0.002	−0.05; 0.05	0.935
Birth sex (M%)	55.9	41.2	1.81	1.08; 3.02	0.023
Weight (g)	3006.6 ± 637.4	3175.3 ± 590.7	−168.7	−315.7; −21.8	0.025
Weight percentile	44.3 ± 31.5	48.3 ± 29.4	−4.04	−11.4; 3.34	0.282
LGA (%)	6.9	10.6	0.62	0.25; 1.54	0.303
Macrosomia (%)	2.9	4.9	0.58	0.15; 2.19	0.417
Low birth weight (%)	20.2	11.5	1.95	1.01; 3.77	0.045
SGA (%)	17.6	8.9	2.19	1.1; 4.52	0.030
	**Asian group** **(*n* = 21)**	**European group** **(*n* = 43)**	**Mean** **Difference/** **Odds ratio**	**95% CI**	***p*-value**
Adverse perinatal outcome ^††^ (%)	22.9	18.3	1.33	0.72; 2.45	0.361
NICU admission (%)	7	6.4	1.09	0.25; 4.82	0.904
Respiratory distress (%)	32.3	14.8	2.74	0.95; 7.93	0.058
Phototherapy (%)	23.8	16.3	1.61	0.44; 5.84	0.469
Hypoglycemia (%)	9.5	23.3	0.64	0.18; 2.23	0.479
Hypocalcemia (%)	14.3	18.6	0.73	0.17; 3.10	0.670

Wks: weeks; LGA: large for gestational age; SGA: small for gestational age; NICU: neonatal intensive care unit. ^†^ Delivery complications: composite of preterm birth (<37 weeks), assisted vaginal delivery, emergency cesarean. ^††^ Adverse perinatal outcome: composite of neonatal death, admission to neonatal intensive care unit, abnormal Apgar score (<7 at 1 min or 5 min), respiratory complications (oxygen therapy, assisted ventilation, intubation, respiratory acidosis, respiratory distress), phototherapy for neonatal jaundice, hypoglycemia, hypocalcemia.

**Table 5:** Delivery complications: multivariable regression analysis.

Variable	OR	95% CI	*p*-Value
Age	1.02	0.96–1.10	0.446
Pre-pregnancy BMI	0.95	0.89–1.01	0.120
Asian ethnicity	2.19	1.11–4.32	0.023
Multiparity	0.62	0.31–1.23	0.174
Gestational hypertension	0.76	0.23–2.54	0.655
Weight gain	1.02	0.97–1.08	0.394
Week at GDM diagnosis	0.98	0.92–1.02	0.232

OR: Odds ratio; CI: confidence interval; BMI: body mass index; GDM: gestational diabetes mellitus.

**Table 6:** SGA infant: multivariable regression analysis.

Variable	OR	95% CI	*p*-Value
Age	1.01	0.94–1.09	0.613
Gestational Hypertension	0.41	0.09–1.80	0.254
Asian ethnicity	2.36	1.08–5.15	0.031
Multiparity	0.92	0.40–2.10	0.857

OR: Odds ratio; CI: confidence interval.

## Data Availability

The data presented in this study are available on request from the corresponding author (the data are not publicly available due to ethical restrictions).

## Abbreviations

The following abbreviations are used in this manuscript:

BMI	Body Mass Index
CI	Confidence Interval
DBP	Diastolic Blood Pressure
FPG	Fasting Plasma Glucose
GDM	Gestational Diabetes Mellitus
HbA1c	Glycated Hemoglobin
LGA	Large for Gestational Age
NICU	Neonatal Intensive Care Unit
OGTT	Oral Glucose Tolerance Test
OR	Odds Ratio
PG	Plasma Glucose
SBP	Systolic Blood Pressure
SD	Standard Deviation
SGA	Small for Gestational Age
T2D	Type 2 Diabetes
